# Factors influencing HIV knowledge among Indian men: A cross-sectional study

**DOI:** 10.1371/journal.pone.0327411

**Published:** 2025-07-23

**Authors:** Jenica Barnwal, Dilwar Hussain

**Affiliations:** 1 Health Services Management, School of Health Systems Studies, Tata Institute of Social Sciences, Mumbai, India; 2 Centre for the Study of Regional Development, Jawaharlal Nehru University, New Delhi, India; Christian Medical College, INDIA

## Abstract

Human Immunodeficiency Virus (HIV) is one of the critical global health issues, posing severe risks due to its ability to weaken the immune system progressively. Without a cure or effective vaccine, HIV remains a serious health threat in developing countries, especially in South Asia, sub-Saharan Africa, and countries such as India. This study explores the socio-economic and demographic determinants of comprehensive knowledge of HIV among Indian men aged 15–54 years. The study used descriptive statistics and binary logistic regression models to examine the predictors of comprehensive knowledge of HIV among men using the latest round of the National Family Health Survey data, 2019−21 (NFHS-5). Results indicate that comprehensive knowledge of HIV was more prevalent among non-adolescents and was positively associated with being unmarried, educated, wealthier, and residing in urban areas. Logistic regression models revealed that men with higher education were nearly three times more likely to have comprehensive knowledge of HIV than those without formal education. Furthermore, men with full mass media exposure, residing in the Western and North-Eastern regions of India, working in the service sector, and belonging to the richest wealth quintile were significantly more likely to possess comprehensive knowledge of HIV. These findings highlight the importance of targeted interventions focusing on education, economic empowerment, and media outreach to address disparities in HIV awareness among men across different socio-economic and demographic backgrounds in India.

## Introduction

Communicable diseases have long been a significant public health issue, and one of the most pressing emerging threats is Human Immunodeficiency Virus (HIV). HIV undermines the immune system by attacking CD4 cells, important white blood cells that help defend the body against infections [1]. As there is currently no complete cure for HIV, it remains a serious and life-threatening disease in the modern era [2]. Globally, HIV has imposed a considerable mortality burden, causing approximately 1.3 million deaths in 2010, though this number declined to about 630,000 by 2023 [3]. However, developing regions continue to bear a disproportionate share of HIV cases due to limited health literacy, lack of awareness and inadequate healthcare access. Although the United Nations Political Declaration on HIV has made significant strides in addressing the epidemic [4], HIV continues to be a major challenge in low and middle income countries of South Asia and sub-Saharan Africa [5].

India faces a double burden of disease, contending with persistent infectious diseases such as HIV, while simultaneously witnessing a rise in non-communicable diseases. Despite progress, the country continues to have the world’s third-largest HIV epidemic [6]. Approximately 2.3 million people were living with HIV in India, with an adult prevalence of around 0.22% by 2021 [7]. Among them, men had a higher HIV prevalence of 0.24% compared to 0.20% among women. According to the National AIDS Control Organization (NACO), this number increased slightly in 2023, with an estimated 2.5 million individuals affected [8]. The risk of HIV acquisition remains concentrated among specific populations, including people who use drugs, sex workers, men who have sex with men (MSM), transgender individuals, and those from economically disadvantaged backgrounds [9–13]. Given the slight increase in prevalence, it is evident that dissemination of knowledge about HIV is pertinent, particularly for men in high-risk populations. Access to accurate HIV-related information can empower men in these vulnerable groups to adopt protective behaviours and reduce their risk of infection. However, acquiring such knowledge depends on several factors, such as socio-economic status, regional disparities, and cultural barriers, which can significantly impact men’s willingness to seek out or discuss HIV information [14,15]. Approximately 87% of HIV cases in India are attributed to sexual transmission, with the remaining cases due to blood transfusions and intravenous drug use [16]. Intravenous drug use, is the primary mode of HIV transmission in the north-eastern states, which report the highest HIV prevalence in the country [17]. Therefore, enhancing HIV knowledge and awareness across India’s diverse population is a critical public health priority. Research has demonstrated that dissemination of accurate information about HIV is one of the most effective strategies to prevent and control its spread globally [18]. In line with this, Sustainable Development Goal (SDG) 3.3 explicitly calls for the ending epidemics such as HIV by 2030, emphasizing that increasing awareness and knowledge about HIV is essential to achieving this target [19].

A wealth of studies in India have investigated HIV knowledge across different population groups, employing varied sample sizes. Much of this research has primarily focused on women [13,20–24], students [25–28], youth [29,30], adolescents [31], teachers [32], and men who have sex with men [33,34]. Some studies have also examined HIV knowledge among Indian adults, including both women and men [35–37]. However a recent study aimed at identifying the proportion and predictors of comprehensive knowledge of HIV among Indian adults [38], combined male and female data, limiting the ability to discern gender-specific determinants. Given that knowledge-related factors may differ by gender, a focused analysis on men is crucial. The study by Pal et al. (2024 highlights the heightened vulnerability of certain male populations to HIV, emphasizing the role of social stigma, violence, healthcare barriers and most importantly knowledge about HIV in shaping their risk. Their findings reinforce the need for targeted interventions to address structural inequalities and gaps in HIV prevention [39]. Following this, the present study contributes to the literature by identifying key determinants of HIV knowledge among men in India.

Despite the critical role of awareness in HIV prevention, research focusing solely on men remains limited and underexplored. This study seeks to bridge that void by analyzing the factors influencing HIV knowledge among men aged 15–54, using the most recent data from the National Family Health Survey (NFHS-5), a nationally representative dataset. By incorporating novel variables not previously examined in similar studies [13,38], this research provides new insights into the socio-demographic determinants of HIV awareness and helps identify different regions with significant knowledge gaps and informs the development of more targeted and effective HIV education interventions.

### Data source & methodology

This study utilizes data from the NFHS-5 (2019–21), a nationally representative survey collecting detailed demographic, socioeconomic, and health information. NFHS-5 employed a stratified two-stage sampling method and included all 707 districts across 28 states and 8 union territories. The survey achieved a response rate of 97% among women and 92% among men [40]. For the present study, a substantial sample of 84,482 men aged 15–54 years was included.

### Outcome variable

The outcome variable of the study is comprehensive knowledge of HIV. according to NFHS, comprehensive knowledge of HIV is the combination of knowledge about various aspects related to HIV prevention and transmission. To evaluate comprehensive knowledge of HIV, men aged 15–54 who reported being aware of HIV were asked five questions: (a) Does condom use during intercourse prevent HIV transmission? (b) Can having one uninfected sexual partner prevent HIV transmission? (c) Can HIV be transmitted through mosquito bites? (d) Can sharing food with an infected person transmit HIV? and (e) Can a healthy-looking person have HIV?

Respondents were classified to have comprehensive HIV knowledge if they answered all five questions correctly [40]. Each correct answer was coded as 1, and each incorrect response as 0. For the purpose of analysis, a composite binary variable was constructed: individuals who answered all five items correctly were coded as ‘1’ (indicating comprehensive knowledge about HIV), while those who answered one or more questions incorrectly were coded as ‘0’, (indicating lack of comprehensive knowledge about HIV).

### Predictor variables

In line with previous studies [13,38,41–43], the present study considered various predictor variables, including age, marital status, educational level, history of HIV testing, place of residence, religion, social category, mass media exposure, and household wealth status. Additionally, occupation and geographical region were also included. A detailed description of the variable categorization is presented in Table 1.

**Table 1:** Description of the variable used in the study.

Variables	Description
Age group (Adolescent vs. Non-Adolescent)	The age was categorized into two groups: ‘Adolescents’ (aged 15–19), (0), and ‘non-adolescents’ (aged 20–54), (1).
Marital Status	Marital status was categorized as: ‘Never Married’ (0), ‘Married’ (1), and ‘Others’ (including Widowed, Divorced, and Separated) (2).
Education level	Education level was categorized into four groups- ‘no education’ (0), ‘primary’ (1), ‘secondary’ (2), and ‘higher’ (3)
History of HIV testing	History of HIV testing was categorized into ‘Yes’ (0) and ‘No’ (1)
Occupation	Occupation was categorized based on employment type into five groups: ‘not working’ (0), ‘service’ (1), ‘agriculture’ (2), ‘labour’ (3), and ‘others’ (4).
Place of Residence	Place of Residence was categorised into- ‘Urban’ (0), ‘Rural’ (1)
Social groups	Social groups were divided into 4 categories- ‘Scheduled Caste’ (0), ‘Scheduled Tribe’ (1), ‘Other Backward Classes’ (2), ‘Others’ (General) (3),
Religion	Religion was categorized into: religion: Hindu (0), Muslim (1), and Others (2). All other religious groups besides Muslims and Hindus are included in the others.
Geographical Region	In order to construct the variable, Indian states were grouped into 6 categories. ‘Northern’ (0) includes Jammu & Kashmir, Ladakh, Himachal Pradesh, Punjab, Rajasthan, Haryana, Uttarakhand, Chandigarh (Union Territory – UT) and Delhi; ‘Central’ (1) includes the states of Uttar Pradesh, Madhya Pradesh and Chhattisgarh; ‘Eastern’ (2) includes the states of Bihar, Jharkhand, West Bengal and Odisha; ‘Western’ (3) includes the states of Gujarat, Maharashtra, Goa and UTs of Dadra & Nagar Haveli and Daman & Diu; ‘Southern’ (4) includes the states of Kerala, Karnataka, Andhra Pradesh, Tamil Nadu and the UTs of Andaman & Nicobar Islands, Pondicherry and Lakshadweep); ‘North-Eastern’ (5) includes the states of Sikkim, Assam, Meghalaya, Manipur, Mizoram, Nagaland, Tripura, and Arunachal Pradesh.
Mass Media exposure	Exposure to mass media was defined as combined access to newspaper, radio, and television at least once a week, measured at the household level. This variable was categorized as: ‘No Exposure’ (0), ‘Partial Exposure’ (1), and ‘Full Exposure’ (2).
Household wealth status	The wealth index is a composite index of household amenities and assets; it indicates the socioeconomic condition of a household. In NFHS-5, every household is given a score based on the number of consumer goods they own. A total of 33 assets and housing characteristics were taken into consideration to prepare a factor score using Principal Component Analysis. Thereafter this factor score was divided into five equal categories, − ‘poorest’ (0), ‘poorer’ (1), ‘middle’ (2), ‘richer’ (3), and ‘richest’ (4), each with 20% of the population.

### Statistical analysis

Descriptive statistics were employed to analyze participant distribution across main predictors and outcome variables. Bivariate analysis, using weighted percentages and Pearson’s chi-square tests, assessed the association between comprehensive HIV knowledge and independent variables. Sampling weights were applied to ensure nationally representative estimates. To check for multicollinearity, the variance inflation factor (VIF) was examined; all variables had VIF values below 10, indicating no significant multicollinearity.

Subsequently, three models of binary logistic regression were incorporated to examine the association between adolescent status and comprehensive knowledge of HIV among men. Model 1 presented the crude association between adolescent status and the outcome variable. Model 2 adjusted for individual-level factors, including marital status, level of male education, and HIV testing. Model 3 further incorporated socio-economic variables were incorporated, including male occupation, caste, and religion. The final model included economic, geographical region, and media exposure

In addition, a coefficient plot was used to understand the individual predictor effects in the final model. The regression results were presented as estimated adjusted odds ratios (AOR) with 95% confidence intervals (CI), with a significance level set at p < 0.05. All statistical analyses were performed using STATA version 13.1.

### Ethics declarations

This study used secondary data drawn from the National Family Health Survey 2019−21, which is available in the public domain. The ethical approval for NFHS-5 (2019–21) was obtained from the International Institute for Population Sciences (IIPS) in Mumbai. Furthermore, the ICF International Review Board (IRB) conducted a review of the survey and granted ethical approval. The datasets are publicly available, and there is no identifiable information about the survey participants. The datasets are freely available from the Demographic Health Survey (DHS) program at https://www.dhsprogram.com/data/available-datasets.cfm. Therefore no separate ethical approval was required to conduct this study.

## Results

The association between comprehensive knowledge of HIV and various socio-economic and demographic characteristics among men aged 15–54 years is outlined in Table 2. Comprehensive knowledge of HIV was higher among non-adolescents as compared to adolescents and significantly associated with marital status (p < 0.001), with unmarried men showing slightly higher knowledge than married men. Both education and household wealth were positively associated with knowledge levels. Among those tested for HIV, 47% of men had comprehensive knowledge. Additionally, men with full mass media exposure were significantly more informed than those with partial or no exposure.

**Table 2:** Sample distribution and comprehensive knowledge of HIV by background characteristics of the study population, India, NFHS-5, 2019−21.

Background Characteristics	Sample size	Comprehensive knowledge of HIV (%)	P-value
**Age-group** (Adolescent vs. Non-Adolescent)			<0.001
Adolescent	13,274	33.56	
Non-adolescent	71,208	38.05	
**Marital status**			<0.001
Never married	30,473	37.92	
Currently married	52,752	37.22	
Others	1,257	29.41	
**Educational level**			<0.001
Illiterate	9,384	22.1	
Primary	9,071	25.27	
Secondary	49,659	37.4	
Higher	16,368	51.13	
**History of HIV testing**			<0.001
No	76,506	36.22	
Yes	7,976	47.36	
**Occupation**			<0.001
Not working	9,973	39.17	
Service	10,448	45.66	
Agriculture	18,332	30.7	
Labour	12,762	35.25	
Others	2,220	35.13	
**Place of residence**			<0.001
Urban	22,638	44.06	
Rural	61,844	33.51	
**Geographical Region**			<0.001
Western	9,668	47.53	
Northern	18,214	42.34	
Central	19,608	28.26	
Eastern	12,107	27.26	
Southern	13,471	39.63	
North-east	11,414	36.29	
**Social group**			<0.001
Scheduled Caste (SC)	18.78	33.62	
Scheduled Tribe (ST)	18.05	30.2	
Other Backward Class (OBC)	39.34	38.17	
Others	23.82	40.92	
**Religion**			<0.001
Hindu	64,762	37.39	
Muslim	9,956	31.83	
Others	9,764	52.52	
**Mass media exposure**			<0.001
No exposure	8,469	18.98	
Partial exposure	20,271	27.91	
Full exposure	55,742	42.97	
**Wealth index**			<0.001
Poorest	15,455	22.74	
Poorer	18,171	29.76	
Middle	18,051	34.66	
Richer	17,292	42.66	
Richest	15,513	51.62	
**Total**	**84,482**	**36.39**	

Furthermore, Comprehensive knowledge of HIV was significantly higher among urban men, those with higher education (50% vs. 22% with no education), and men from the Western region (48%) compared to the Eastern region (28%) in India. Service sector employees and those classified under ‘other’ social and religious groups also showed greater knowledge.

Model 1 included age as the primary predictor. Model 2 added marital status, educational attainment, and HIV testing history. Model 3 further incorporated occupational status (Table 3). Finally, Model 4 included geographical region, social group, religion, mass media exposure, place of residence, and household wealth quintile. In Model 1, non-adolescent men were significantly more likely to have comprehensive knowledge of HIV than adolescent men (AOR: 1.20, 95% CI: 1.16–1.25). Model 2 revealed that men with secondary education were more than twice as likely (AOR: 2.14, 95% CI: 2.03–2.26), and those with higher education nearly three times as likely (AOR: 3.28, 95% CI: 3.09–3.48), to possess comprehensive knowledge compared to illiterate men. Additionally, men who had ever been tested for HIV were 47% more likely to have comprehensive knowledge than those who had never been tested (AOR: 1.47, 95% CI: 1.41–1.55).

**Fig 1:**
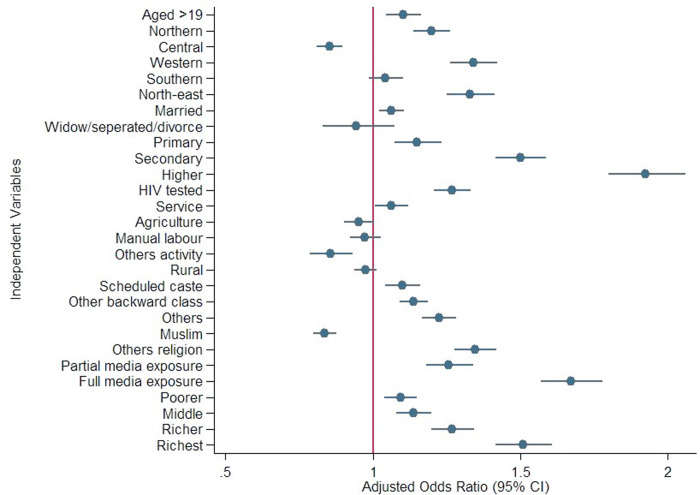
Coefficient plot of odds ratios for comprehensive HIV knowledge by socio-demographic predictors.

Model 3, which included occupational status, showed that men employed in the service sector had greater odds of comprehensive knowledge (AOR: 1.11, 95% CI: 1.05–1.17). In the final model, men from the Western (AOR: 1.33, 95% CI: 1.26–1.42) and North-Eastern (AOR: 1.32, 95% CI: 1.24–1.41) regions were more likely to possess comprehensive knowledge compared to those from the Eastern region. Men belonging to OBC (AOR: 1.13, 95% CI: 1.08–1.18) and other categories (AOR: 1.22, 95% CI: 1.16–1.28) had higher odds than SC. Muslim men had lower odds (AOR: 0.83, 95% CI: 0.79–0.87). Men fully exposed to mass media were 67% more likely to have comprehensive knowledge (AOR: 1.67, 95% CI: 1.56–1.78). Moreover, wealth status was positively associated with knowledge, with men in the wealthiest quintile having 50% greater odds of comprehensive knowledge than those in the poorest quintile (AOR: 1.50, 95% CI: 1.41–1.60). The coefficient plot (Fig 1) also shows the significant impact of different socio-economic determinants on HIV knowledge, as revealed by the logistic regression in Model 4.

**Table 3:** Factors influencing HIV knowledge among men (aged 15−54 years) in India, NFHS-5, 2019−21.

Predictors	Model 1	Model 2	Model 3	Model 4
**Age-group** (Adolescent vs. Non-Adolescent)				
Adolescent	Ref	Ref	Ref	Ref
Non adolescent	1.2 (1.16-1.25) ***	1.15 (1.09-1.21) ***	1.14(1.08-1.20) ***	1.10 (1.04-1.16) ***
**Marital status**				
Never married		Ref	Ref	Ref
Currently married		1.04 (1.00-1.08) ***	1.05(1.01-1.10) **	1.06 (1.02-1.10) **
Others		0.91 (0.80-1.03)	0.89(0.79-1.02)	0.94 (0.82-1.07)
**Educational level**				
Illiterate		Ref	Ref	Ref
Primary		1.30(1.22-1.39) ***	1.26 (1.18-1.35) ***	1.15 (1.07-1.23) ***
Secondary		2.14 (2.03-2.26) ***	1.96 (1.86-2.07) ***	1.49 (1.41-1.58) ***
Higher		3.28(3.09-3.48) ***	2.75 (2.58-2.93) ***	1.92 (1.79-2.05) ***
**History of HIV testing**				
No		Ref	Ref	Ref
Yes		1.47(1.41-1.55) ***	1.36 (1.30-1.43) ***	1.26 (1.20-1.33) ***
**Occupation**				
Not working			Ref	Ref
Service			1.11 (1.05-1.17) ***	1.06 (1.00-1.11)
Agriculture			0.89 (0.84-0.94) ***	0.95 (0.90- 1.00)
Labour			0.96 (0.91-1.01)	0.97 (0.92-1.02)
Others			0.88 (0.81-0.95) **	0.85 (0.78-0.93) ***
**Place of residence**				
Urban				Ref
Rural				0.97 (0.93-1.01)
**Geographical Region**				
Western				Ref
Northern				1.19 (1.13-1.26) ***
Central				0.85 (0.80-0.89) ***
Eastern				1.33 (1.26-1.42) ***
Southern				1.04 (0.98-1.01)
North-east				1.32 (1.24-1.41) ***
**Social group**				
Scheduled Caste				Ref
Scheduled Tribe				1.09 (1.04-1.15) **
Other Backward Class				1.13 (1.08-1.18) ***
Others				1.22 (1.16-1.28) ***
**Religion**				
Hindu				Ref
Muslim				0.83 (0.79-0.87) ***
Others				1.34 (1.27-1.42) ***
**Mass media exposure**				
No exposure				Ref
Partial exposure				1.25 (0.79-0.87) ***
Full exposure				1.67 (1.15-1.78) ***
**Wealth index**				
Poorest				Ref
Poorer				1.09 (1.03-1.33) **
Middle				1.13 (1.07-1.19) ***
Richer				1.26 (1.19-1.34) ***
Richest				1.50 (1.41-1.60) ***

Note: Ref: reference group, ***: p < 0.01, and **: p < 0.05. AOR: Adjusted odds ratios for the outcome variable, 95% confidence intervals

## Discussion

In assessing the knowledge of HIV among men aged 15–54 years in India, the study found that 36.3% of men had comprehensive knowledge of HIV. The study further identified several significant predictors of HIV knowledge, including age, religion, history of HIV testing, and, most notably, exposure to mass media. Regional disparities were evident, with men from Western India showing higher levels of knowledge compared to those from the Eastern region. Additionally, men in higher wealth quintiles were more likely to have a comprehensive knowledge of HIV.

We found that adolescent respondents demonstrated lower levels of HIV knowledge compared to non-adolescents. Adolescents represent a particularly vulnerable age group, as their limited knowledge of HIV may impede their ability to adopt protective behaviours, placing them at higher risk for engaging in high-risk sexual practices. These findings align with results from studies conducted in Iran [44], Bangladesh [42], India [45,46], and Nigeria [41]. Another reason could be the limited access to reliable information sources, as well as inadequate school-based HIV education, often due to a shortage of skilled teachers [47].

The likelihood of having comprehensive HIV knowledge increased with higher education, consistent with findings from several African and Asian countries [13,46,48–51]. People with higher levels of education are more likely to obtain, comprehend, and act upon health information, including public health campaigns and school-based interventions. This may be attributed to greater health awareness and a more proactive approach to obtaining health information among more educated individuals. Young people gain essential HIV knowledge through school-based interventions, and education on sexual and reproductive health is strongly linked to better understanding. Additionally, higher literacy enhances individuals’ ability to comprehend public health campaigns and fostering discussions about HIV prevention [13,51]. Consistent with previous findings in India [46], Bangladesh [52], USA [53,54] and Canada [43], the study showed that residents in urban areas had higher HIV knowledge. Urban residents are generally more likely to have access to education, leading to greater self-efficacy, awareness, and adherence to healthy behaviours. Access to health information, especially through mass media like television and newspapers [43] is more prevalent in urban areas, which contributes to increased HIV awareness, especially in developing nations [55,56].

A positive association between HIV testing and HIV knowledge among men is supported by research from Ethiopia [57], Kenya [58], Nigeria [59] and India [13,46]. This relationship may be attributed to the dissemination of key information during pre-test and post-test counselling, which reinforces awareness of HIV prevention and transmission [60,61]. The study also found that men belonging to religious groups categorized as ‘others’ such as Christianity, Jainism, Buddhism, and Zoroastrianism were more likely to possess comprehensive HIV knowledge compared to those identifying as Hindu or Muslim. Although these group represent a smaller proportion of the population in the country, their relatively higher literacy rates contribute to better access to HIV-related information and health education [62]. Furthermore the higher concentration of Christians in North-Eastern India, a region with a higher prevalence of HIV, suggests that local administration and socio-cultural organizations have effectively promoted HIV education and awareness [7].

In line with studies from Sub-Saharan [52,63,64] and South Asian countries [13,65],full exposure to mass media was associated with a higher likelihood of having comprehensive knowledge of HIV among men. Mass media through television, radio, and newspapers has become an important source of HIV information, often reaching large segments of the population at a low cost [66,67]. These channels are effective in spreading knowledge and encouraging healthier behaviours. In many developing countries, mass media is a key tool in HIV awareness campaigns because of its broad reach and affordability. Unlike formal education, which may not be accessible to everyone, mass media has the potential to reach individuals regardless of their location, education level, or income. Television and radio, for instance, are commonly available even in rural areas, bridging the information gap between urban and rural populations. Additionally, social networks contribute to the spread of HIV-related information, particularly among middle-aged groups. A study from Bangladesh further supports this, showing that reading newspapers was linked to improved knowledge about HIV, highlighting the importance of education and information spread through accessible media sources [51].

One of the crucial findings of this study is the higher likelihood of comprehensive HIV knowledge among men from Western and North-eastern India compared to those from Eastern India. This disparity can likely be explained by the focused efforts in these regions to raise awareness. For instance, in Mizoram, the Mizoram State AIDS Control Society (MSACS), established in 1998, has been spearheading HIV prevention and awareness efforts, following the creation of the State AIDS Cell in 1992 [7]. In a similar effort, the West Bengal State AIDS Prevention and Control Society has undertaken consistent initiatives to educate the public about HIV and ways to prevent it [68]. In addition, wealth quintile was found to be positively associated with HIV knowledge among men. This finding is similar to previous studies [35,57,69,70] Implementing HIV awareness in all settings is essential for ensuring people receive accurate information and dispelling misconceptions. Expanding access to HIV counselling and testing facilities can substantially improve HIV knowledge among Indian men. Comprehensive knowledge is crucial for promoting protective behaviours, reducing myths, and combating stigma around HIV. Identifying the determinants of HIV knowledge can help uncover cultural and societal barriers that limit access to accurate information. Effective educational programs, including behaviour change strategies and mass media campaigns, are needed to enhance awareness, especially in rural areas [51,71,72]. India has made substantial progress in its response to HIV. Although HIV prevalence has significantly decreased in recent years, continued efforts are crucial for raising awareness about its spread and prevention [73,74]. The National AIDS Control Programme (NACP) plays a central role in this by emphasizing widespread information, education, and communication on HIV prevention (NACO, 2024). Under the ‘Azadi ka Amrit Mahotsav,’ campaigns have promoted awareness and reduced stigma, involving students from hundreds of schools and colleges in awareness activities. The continued focus on education, especially in academic institutions has been instrumental in spreading accurate knowledge and changing public perceptions. The NACP also aims to reduce new HIV infections and AIDS-related deaths from 2010 levels, reflecting successful strides in raising public awareness and improving health outcomes [75].

### Strengths and limitations

One of the important contributions of this study lies in its ability to identify regional and social disparities in HIV knowledge, helping to pinpoint specific geographical zones and demographic subgroups where knowledge gaps are most pronounced. By leveraging nationally representative NFHS-5 data, this study not only provides a comprehensive overview but also highlights trends that may inform targeted health communication campaigns and intervention programs. Policymakers and public health stakeholders can use these findings to tailor HIV awareness programs based on regional needs, ensuring that educational efforts reach communities with the highest levels of misinformation or lack of awareness. This study has certain limitations that must be acknowledged. As it relies on secondary data from the National Family Health Survey (NFHS-5), it is subject to potential reporting biases. The cross-sectional nature of the data prevents establishing causal relationships between HIV knowledge and its determinants. Additionally, while the study identifies key predictors of comprehensive HIV knowledge among men, unmeasured confounders such as exposure to HIV campaigns, access to healthcare services, and social media usage may influence the findings which were not collected in NFHS-5. The lack of direct information on high-risk behaviors, such as MSM status, limits a more nuanced understanding of vulnerability. Despite these constraints, the study provides valuable insights into HIV knowledge gaps

## Conclusion

This study highlights the critical importance of obtaining accurate and complete knowledge about HIV and examines the factors influencing such knowledge among Indian men aged 15–54 years. The findings revealed that only one in three adolescent men possesses comprehensive knowledge of HIV, emphasizing the need for targeted awareness efforts for this vulnerable group. Determinants such as marital status, education, HIV testing history, region, wealth, religion, and social category influence their knowledge. Men from Central India were less likely to have comprehensive knowledge, suggesting that government efforts should place additional focus in this geographical pocket. Policies should address knowledge gaps among men, particularly within underprivileged groups. Mass media campaigns and community-based educational efforts should target Muslims, less educated, and unemployed men. Furthermore, expanding access to HIV testing and counselling services, especially for marginalized populations, may further enhance awareness. These efforts are essential not only for improving public health outcomes but also for contributing tothe achievement of Sustainable Development Goal 3.3, which aims to end the AIDS epidemic by 2030.
